# Quantitative Anatomical Comparison of Surgical Approaches to Meckel’s Cave

**DOI:** 10.3390/jcm12216847

**Published:** 2023-10-30

**Authors:** Luca Zanin, Edoardo Agosti, Florian Ebner, Lucio de Maria, Francesco Belotti, Barbara Buffoli, Rita Rezzani, Bernard Hirt, Marco Ravanelli, Tamara Ius, Marco Zeppieri, Marcos Soares Tatagiba, Marco Maria Fontanella, Francesco Doglietto

**Affiliations:** 1 Neurosurgery Unit, Department of Medical and Surgical Specialties, Radiological Sciences and Public Health, University of Brescia, 25123 Brescia, Italyedoardo_agosti@libero.it (E.A.);; 2 Department of Neurological Surgery, Eberhard-Karls University, Tübingen University Hospital, D-72076 Tübingen, Germany; 3 Section of Anatomy and Physiopathology, Department of Clinical and Experimental Sciences, University of Brescia, 25123 Brescia, Italy; 4 Department of Clinical Anatomy, Eberhard-Karls-University, Tübingen University Hospital, D-72076 Tübingen, Germany; 5 Radiology Unit, Department of Medical and Surgical Specialties, Radiological Sciences and Public Health, University of Brescia, 25123 Brescia, Italy; 6 Neurosurgery Unit, Head-Neck and NeuroScience Department, University Hospital of Udine, p.le S. Maria della Misericordia 15, 33100 Udine, Italy; 7 Department of Ophthalmology, University Hospital of Udine, p.le S. Maria della Misericordia 15, 33100 Udine, Italy; 8 Neurosurgery, Fondazione Policlinico Universitario A. Gemelli IRCSS, 00168 Rome, Italy; 9 Neurosurgery, Università Cattolica del Sacro Cuore, 20123 Rome, Italy

**Keywords:** Meckel’s cave, quantitative comparison, skull base surgery, endoscopy, microsurgery, anatomy

## Abstract

Background: Meckel’s cave is a challenging surgical target due to its deep location and proximity to vital neurovascular structures. Surgeons have developed various microsurgical transcranial approaches (MTAs) to access it, but there is no consensus on the best method. Newer endoscopic approaches have also emerged. This study seeks to quantitatively compare these surgical approaches to Meckel’s cave, offering insights into surgical volumes and exposure areas. Methods: Fifteen surgical approaches were performed bilaterally in six specimens, including the pterional approach (PTA), fronto-temporal-orbito-zygomatic approach (FTOZA), subtemporal approach (STA), Kawase approach (KWA), retrosigmoid approach (RSA), retrosigmoid approach with suprameatal extension (RSAS), endoscopic endonasal transpterygoid approach (EETPA), inferolateral transorbital approach (ILTEA) and superior eyelid approach (SEYA). All the MTAs were performed both with 10 mm and 15 mm of brain retraction, to consider different percentages of surface exposure. A dedicated navigation system was used to quantify the surgical working volumes and exposure of different areas of Meckel’s cave (ApproachViewer, part of GTx-Eyes II, University Health Network, Toronto, Canada). Microsurgical transcranial approaches were quantified with two different degrees of brain retraction (10 mm and 15 mm). Statistical analysis was performed using a mixed linear model with bootstrap resampling. Results: The RSAS with 15 mm of retraction offered the maximum exposure of the trigeminal stem (TS). If compared to the KWA, the RSA exposed more of the TS (69% vs. 46%; *p* = 0.01). The EETPA and ILTEA exposed the Gasserian ganglion (GG) mainly in the anteromedial portion, but with a significant 20% gain in exposure provided by the EETPA compared to ILTEA (42% vs. 22%; *p* = 0.06). The STA with 15 mm of retraction offered the maximum exposure of the GG, with a significant gain in exposure compared to the STA with 10 mm of retraction (50% vs. 35%; *p* = 0.03). The medial part of the three trigeminal branches was mainly exposed by the EETPA, particularly for the ophthalmic (66%) and maxillary (83%) nerves. The EETPA offered the maximum exposure of the medial part of the mandibular nerve, with a significant gain in exposure compared to the ILTEA (42% vs. 11%; *p* = 0.01) and the SEY (42% vs. 2%; *p* = 0.01). The FTOZA offered the maximum exposure of the lateral part of the ophthalmic nerve, with a significant gain of 67% (*p* = 0.03) and 48% (*p* = 0.04) in exposure compared to the PTA and STA, respectively. The STA with 15 mm of retraction offered the maximum exposure of the lateral part of the maxillary nerve, with a significant gain in exposure compared to the STA with 10 mm of retraction (58% vs. 45%; *p* = 0.04). The STA with 15 mm of retraction provided a significant exposure gain of 23% for the lateral part of the mandibular nerve compared to FTOZA with 15 mm of retraction (*p* = 0.03). Conclusions: The endoscopic approaches, through the endonasal and transorbital routes, can provide adequate exposure of Meckel’s cave, especially for its more medial portions, bypassing the impediment of major neurovascular structures and significant brain retraction. As far as the most lateral portion of Meckel’s cave, MTA approaches still seem to be the gold standard in obtaining optimal exposure and adequate surgical volumes.

## 1. Introduction

The trigeminal cave, or Meckel’s cave, originally described by Johann Friedrich Meckel the Elder in 1748, is a cerebrospinal-fluid-containing dural pouch in the medial portion of the middle cranial fossa and adjacent to the cavernous sinus [1]. It opens to the posterior cranial fossa and houses the trigeminal ganglion (TG). Its deep location, the presence of the temporal lobe, and the anatomical proximity to vital neurovascular structures make its surgical access challenging [2].

Several microsurgical transcranial approaches (MTAs) to Meckel’s cave have been described over time, but a common opinion among authors is still lacking as to which approach can quantitatively offer the best exposure. Conversely, the choice of a surgical approach often relies on personal preference, the level of comfort of the surgeon, and the overall goals of the procedure (e.g., simple debulking for mass effect release, radical resection, etc.). Moreover, with the recent introduction of endoscopic endonasal approaches and endoscopic transorbital approaches (ETOAs), new surgical trajectories to Meckel’s cave have been proposed.

Although clinical comparative analyses of different surgical approaches to Meckel’s cave are available [3,4], they often include a small number of patients of single-center case series or do not consider all the commonly used surgical approaches to Meckel’s cave. Therefore, the aim of this study is to perform a quantitative anatomical comparison of the most used surgical approaches to Meckel’s cave, describing surgical volumes and areas of exposure.

## 2. Materials and Methods

Cadavers were obtained from the body donation program of the Institute of Anatomy at the University of Brescia. Prior to death, the donors had all given written consent to the use of the body for educational and research purposes. The general use of cadavers for teaching purposes is a common practice and has been widely approved by the University Ethics Board. Formal ethics committee approval for this type of research on cadavers was not required by our University. The research was conducted in full compliance with the ethical guidelines established by our Institutional Review Board. All investigations involving human cadavers were carried out in strict adherence to the ethical principles outlined in the 1964 Declaration of Helsinki and its subsequent revisions.

Of note, the methods of this study were replicated from previous peer-reviewed anatomical studies both from our group and in the literature [5,6,7,8].

### 2.1. Preparation of Specimens and Neuronavigation

A total of 6 alcohol-fixed specimens (12 sides) were dissected. Intracranial arteries were injected with red silicone rubber.

Each specimen underwent a 128-multidetector computed tomography scan (Somatom^®^ Definition Flash, Siemens, Forcheim, Germany). Subsequently, the Digital Imaging and Communications in Medicine (DICOM) records of the CT scans were transferred to a specialized neuronavigation software program (v. 1, GTx-Eyes II Approach Viewer, University Health Network, University of Toronto, Toronto, ON, Canada) [8].

### 2.2. Surgical Approaches to Dissection

The dissections were conducted at the Anatomy Laboratory of the University of Brescia (Italy) and the Anatomy Laboratory of the University of Tubingen (Germany) with the utilization of conventional microsurgical and endoscopic tools from Karl Storz^®^ (Tüttlingen, Germany). To capture and record the intricate details of the microsurgical and endoscopic anatomy, a Leica M320^®^ surgical microscope (Leica Microsystems Srl, Buccinasco, Italy) and a 4 K camera head from Olympus^®^ (Segrate, Italy) were employed, respectively.

**Figure 1:**
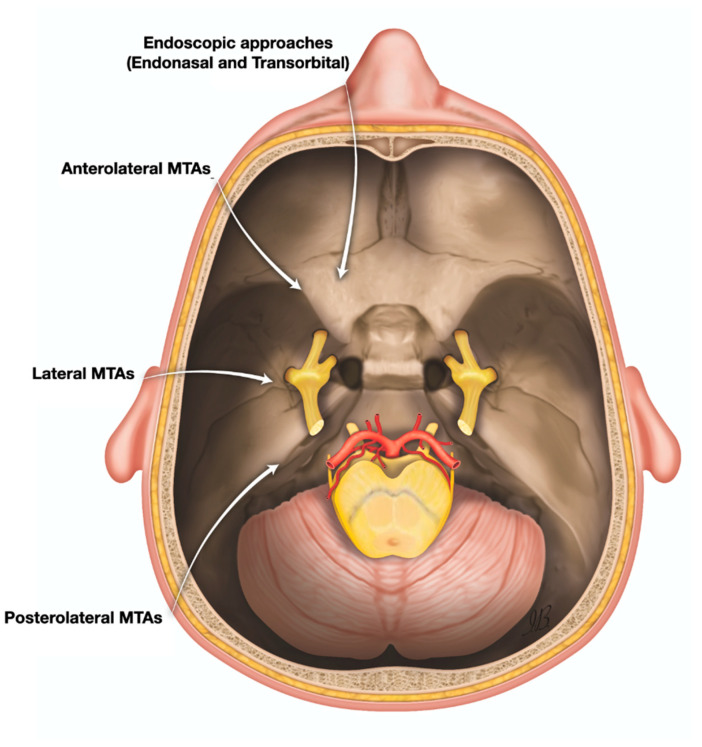
Schematic representation of the surgical approaches performed on each specimen.

Fifteen surgical approaches were performed on each specimen. A schematic representation of these approaches is shown in Figure 1.

The following anterolateral MTAs were investigated:

1. Pterional approach (PTA), according to Yasargil et al. [9], with 10 and 15 mm of retraction;
2. Fronto-temporal-orbito-zygomatic approach (FTOZA) according to Van Furth et al. [10], with 10 and 15 mm of retraction.

The following lateral MTAs were investigated:

1. Kawase approach (KWA), according to Kawase et al. [11], with 10 and 15 mm of retraction;
2. Subtemporal approach (STA), according to Dolenc et al. [12], with 10 and 15 mm of retraction.

The following posterolateral MTAs were investigated:

1. Retrosigmoid approach (RSA) according to Samii et al. [13], with 10 and 15 mm of retraction;
2. Retrosigmoid approach with suprameatal extension (RSAS) according to Samii et al. [5], with 10 and 15 mm of retraction.

The following endoscopic approaches were investigated:

1. Endoscopic endonasal transpterygoid approach (EETPA), according to Agosti et al. [7];
2. Inferolateral transorbital endoscopic approach (ILTEA), according to Ferrari et al. [7];
3. Superior eyelid approach (SEYA), according to Locatelli et al. [14].

As for MTAs, the surgical volumes were quantified with two different retraction degrees (i.e., 10 and 15 mm), to evaluate the exposure advantage as cerebral retraction increases. Brain retraction was kept constant during the quantification with the use of a Greenberg^®^ Retractor System, parallelly positioned at 10 and 15 mm from the sphenoid ridge, middle cranial fossa, and posterior surface of the petrous bone for the anterolateral, lateral, and posterolateral MTAs, respectively [7].

### 2.3. Quantification of the Surgical Corridor

We employed an optical neuronavigation system (Polaris Vicra^®^; NDI, Waterloo, ON, Canada) in conjunction with GTx-Eyes II for the assessment of the maximum surgical volume with optimal maneuverability, termed the “crossing” modality, and the largest exposure achievable with straight instruments, referred to as the “non-crossing” modality [7]. Each modality was evaluated through three data collection iterations.

For MTAs, the height of the surgical corridor was established at the level of the craniotomy, while, for ETOAs, it was set at the orbital rim. In the case of EETPA, the surgical corridor height was aligned with the nasal pyriform aperture.

### 2.4. Surface Rendering and Quantification of the Exposed Area

Meckel’s cave was considered as an open-ended three-fingered glove, enveloping the trigeminal ganglion, the ophthalmic nerve (V1), maxillary nerve (V2), and mandibular nerve (V3) divisions until they reach the correspondent skull base foramina [1,2].

**Figure 2:**
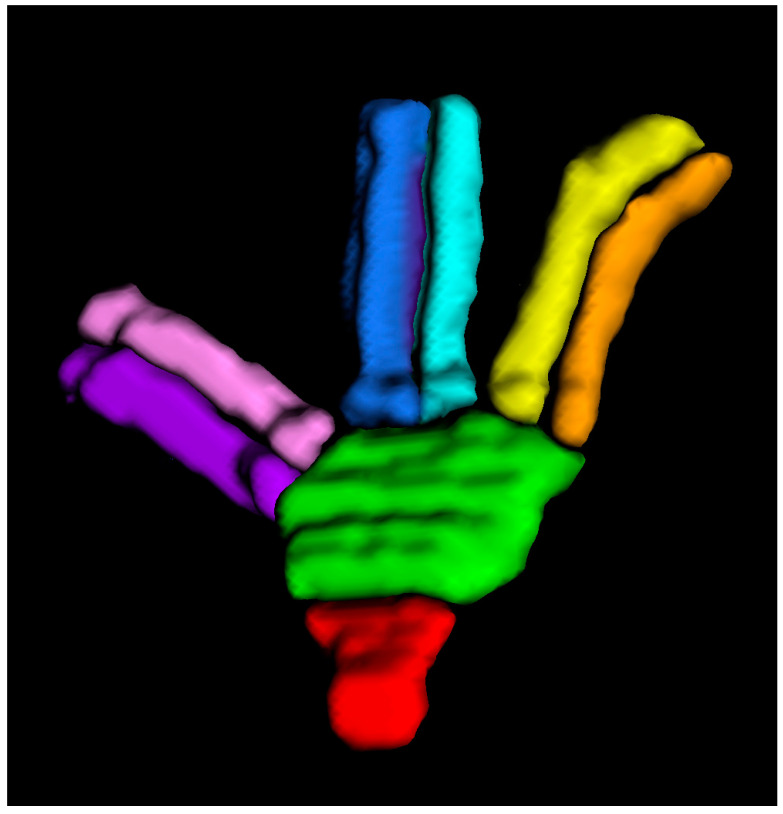
The 8 surfaces of Meckel’s cave that were rendered with ITK-SNAP software from CT scans. Red: trigeminal stem; green: Gasserian ganglion; orange: V1 medial; yellow: V1 lateral; light-blue: V2 medial; blue: V2 lateral; purple: V3 medial; violet: V3 lateral.

Meckel’s cave was divided into 8 surfaces, rendered with the ITK-SNAP software v. 4.0.2 from each CT scan (Figure 2). Dedicated software (Autodesk Meshmixer v. 3.5^®^ and ApproachViewer v. 1), part of GTX-Eyes-II) quantified the percentage value of the exposed area by all approaches for each of the 8 surfaces [7].

### 2.5. Statistical Analysis

The Meckel’s cave exposure and surgical volume of the different approaches were compared using linear mixed models with random intercepts for specimens. The final estimate was expressed as the β coefficient and 95% CI and was calculated using the bootstrap resampling method with 1000-fold replications. Statistical significance was set at *p* < 0.05. All analyses were performed using the STATA^®^ software v. 16.1 (StataCorp^®^ LLC., College Station, TX, USA).

## 3. Results

**Figure 3:**
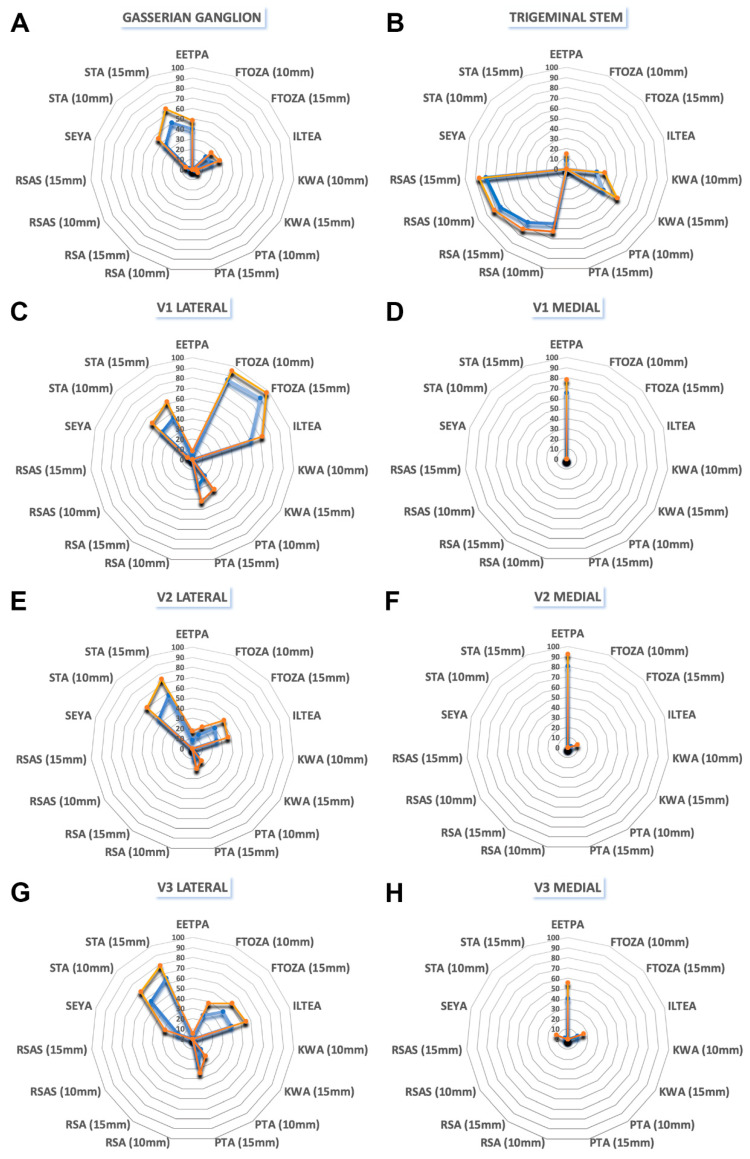
Visual depiction of the average exposed surface area percentages for each surgical approach in relation to Meckel’s cave. Orange line: crossing measurements; blue line: non-crossing measurements. Abbreviations: EETPA, endoscopic endonasal transpterygoid approach; FTOZA, fronto-temporal-orbito-zygomatic approach; ILTEA, infero-lateral transorbital endoscopic approach; RSA, retrosigmoid approach; RSAS, retrosigmoid approach with suprameatal extension; PTA, pterional approach; SEYA, superior eyelid approach; KWA, Kawase approach; STA, subtemporal approach.

**Figure 4:**
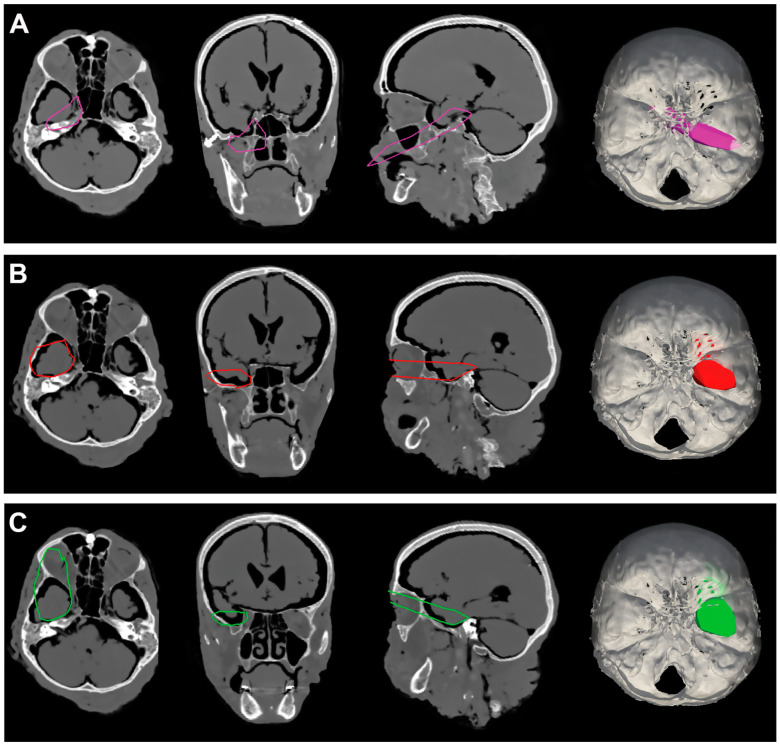
(**A**). Exemplificative screenshot from Approach Viewer of the EETPA. (**B**). Exemplificative screenshot from Approach Viewer of the ILTEA. (**C**). Exemplificative screenshot from Approach Viewer of the SEYA.

**Figure 5:**
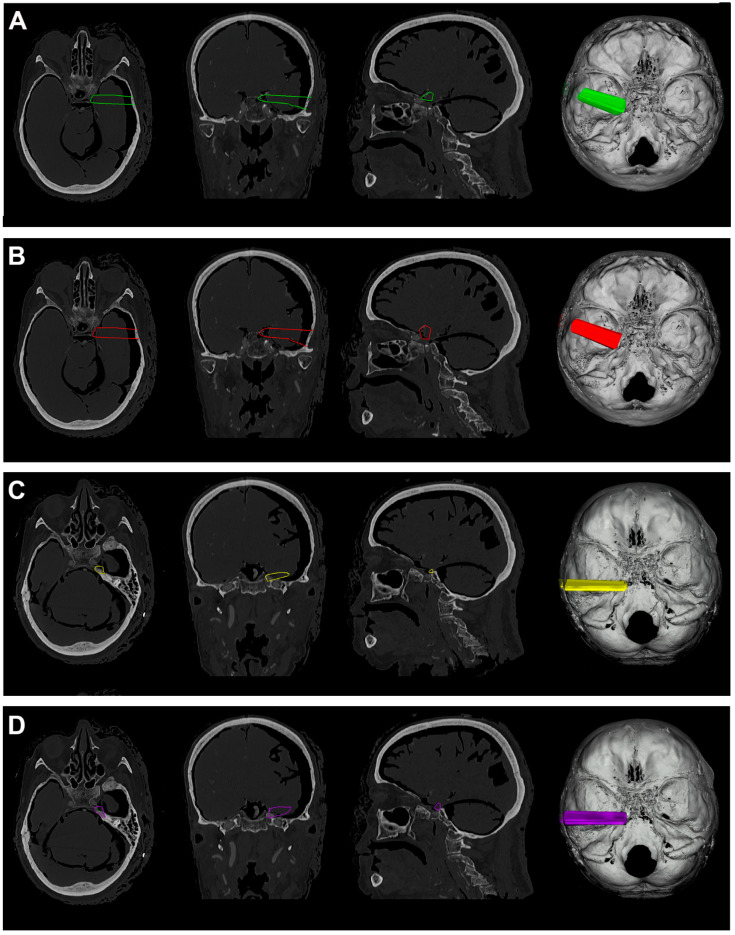
(**A**). Exemplificative screenshot from Approach Viewer of the STA with 10 mm of retraction. (**B**). Exemplificative screenshot from Approach Viewer of the STA with 15 mm of retraction. (**C**). Exemplificative screenshot from Approach Viewer of the KWA with 10 mm of retraction. (**D**). Exemplificative screenshot from Approach Viewer of the KWA with 15 mm of retraction.

**Figure 6:**
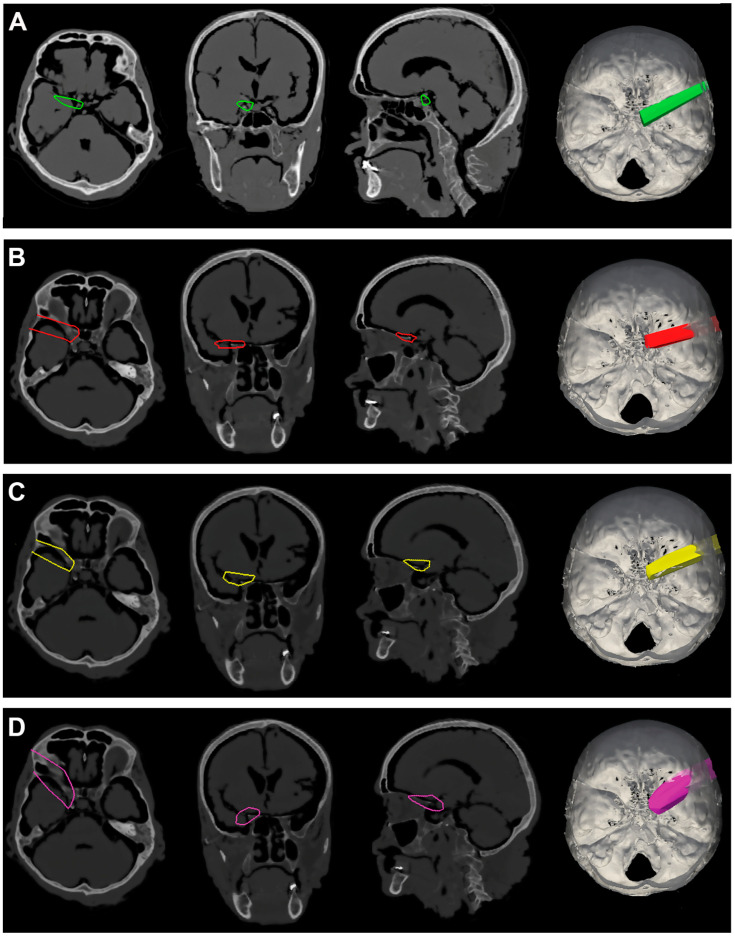
(**A**). Exemplificative screenshot from Approach Viewer of the PTA with 10 mm of retraction. (**B**). Exemplificative screenshot from Approach Viewer of the PTA with 15 mm of retraction. (**C**). Exemplificative screenshot from Approach Viewer of the FTOZ with 10 mm of retraction. (**D**). Exemplificative screenshot from Approach Viewer of the FTOZ with 15 mm of retraction.

**Figure 7:**
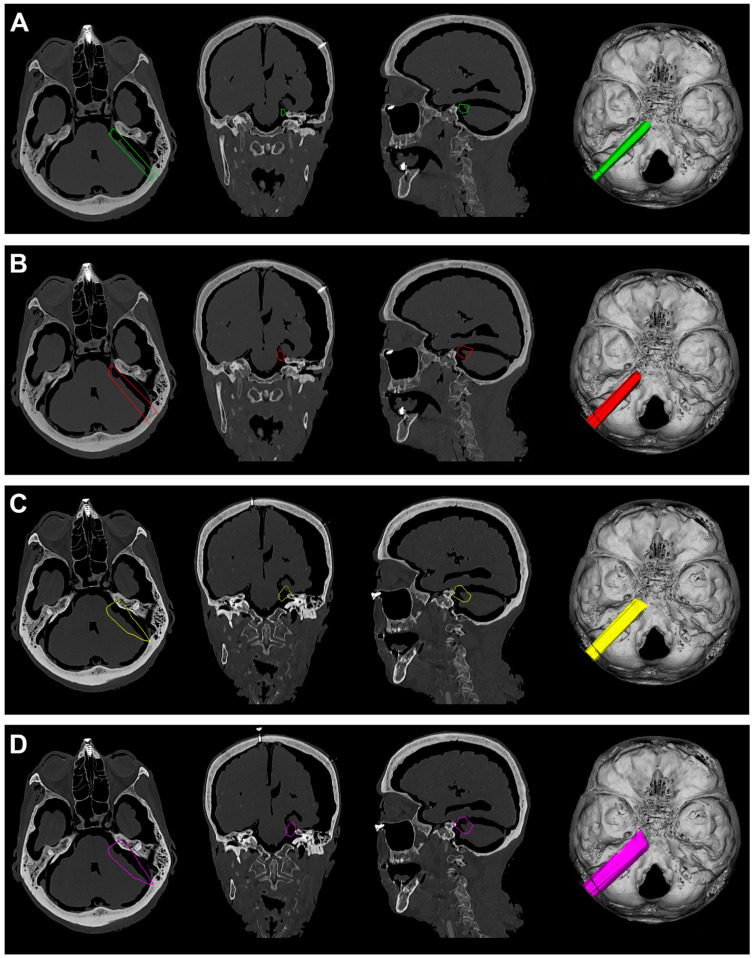
(**A**). Exemplificative screenshot from Approach Viewer of the RSA with 10 mm of retraction. (**B**). Exemplificative screenshot from Approach Viewer of the RSA with 10 mm of retraction. (**C**). Exemplificative screenshot from Approach Viewer of the RSAS with 10 mm of retraction. (**D**). Exemplificative screenshot from Approach Viewer of the RSAS with 15 mm of retraction.

A grand total of 720 intersection data points were gathered through the execution of surgical procedures involving MTAs, EETPA, and ETOAs, all directed towards Meckel’s cave. Detailed breakdowns of the average percentages of the exposed area on each surface of Meckel’s cave, facilitated by each respective surgical approach, can be found in Table 1, Table 2, Table 3, Table 4, Table 5, Table 6, Table 7 and Table 8. A visual representation of these findings is depicted in Figure 3. Additionally, Figure 4, Figure 5, Figure 6 and Figure 7 provide illustrative screen captures from the Approach Viewer for each of the distinct surgical approaches.

### 3.1. Areas of Exposure

#### 3.1.1. Gasserian Ganglion (GG)

The STA with 15 mm of retraction offered the maximum exposure of the GG, with a significant gain in exposure compared to the STA with 10 mm of retraction (50% vs. 35%; *p* = 0.03). The EETPA and ILTEA exposed the GG mainly in the anteromedial portion, but with a significant 20% gain in exposure provided by the EETPA compared to ILTEA (42% vs. 22%; *p* = 0.06). The lowest exposure of the GG was provided by the KWA (2%).

#### 3.1.2. Trigeminal Stem (TS)

The RSAS with 15 mm of retraction offered the maximum exposure of the TS, without any significant gain in exposure compared to the RSAS with 10 mm of retraction (78% vs. 75%; *p* = 0.73). If compared to the KWA, the RSA exposed more of the TS (69% vs. 46%; *p* = 0.01). Neither the anterolateral MTAs nor the ETOAs provided any exposure to this region.

#### 3.1.3. Ophthalmic Nerve (V1): Medial (V1m) and Lateral (V1l) Portions

The V1m is mainly exposed by the EETPA (66%). The FTOZA offered the maximum exposure of the V1l, with a significant gain of 67% (*p* = 0.03) and 48% (*p* = 0.04) in exposure compared to the PTA and STA, respectively. The ILTEA is the endoscopic approach that offers the major exposure (61%) of the V1l. Neither the anterolateral EETPA nor the SEYA provided any significant exposure to this region.

#### 3.1.4. Maxillary Nerve (V2): Medial (V2m) and Lateral (V2l) Portions

The EETPA offered the greatest exposure of the V2m (83%). The STA with 15 mm of retraction offered the maximum exposure of the V2l, with a significant gain in exposure compared to the STA with 10 mm of retraction (58% vs. 45%; *p* = 0.04). The STA with 15 mm of retraction provided a significant exposure gain of 27% and 53% compared to FTOZA and PTA with parity of retraction, respectively. The ILTEA is the endoscopic approach that offers the greatest exposure (29%) of the V2l.

#### 3.1.5. Mandibular Nerve (V3): Medial (V3m) and Lateral (V3l) Portions

The EETPA is the endoscopic approach that offers the maximum exposure of the V3m, with a significant gain in exposure compared to the ILTEA (42% vs. 11%; *p* = 0.01) and the SEY (42% vs. 2%; *p* = 0.01). The STA with 15 mm of retraction offered the maximum exposure of the V3l, without any significant gain in exposure compared to the STA with 10 mm of retraction (65% vs. 57%; *p* = 0.23). The STA with 15 mm of retraction provided a significant exposure gain of 23% compared to FTOZA with 15 mm of retraction (*p* = 0.03). The FTOZA with 15 mm of retraction is the anterolateral MTA that offers the maximum exposure of the V3l, with a significant gain in exposure compared to the PTA with 15 mm of retraction (42% vs. 26%; *p* = 0.04).

### 3.2. Surgical Volumes

**Figure 8:**
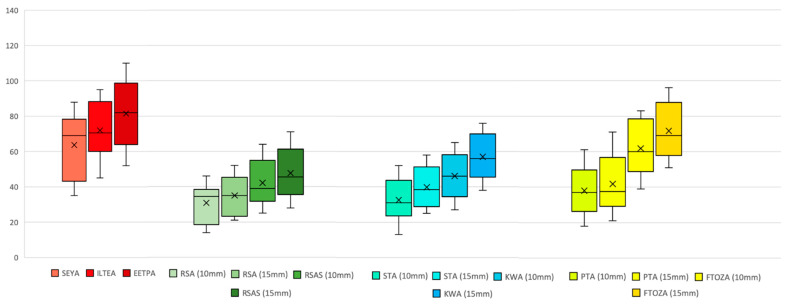
Graphical representation of the minimum, average, maximum, and standard deviation values of the non-crossing volume of each simulated approach. Abbreviations: EETPA, endoscopic endonasal transpterygoid approach; FTOZA, fronto-temporal-orbito-zygomatic approach; ILTEA, infero-lateral transorbital endoscopic approach; RSA, retrosigmoid approach; RSAS, retrosigmoid approach with suprameatal extension; PTA, pterional approach; SEYA, superior eyelid approach; KWA, Kawase approach; STA, subtemporal approach.

The endoscopic methods demonstrated comparable working volumes (EETPA: 84 cm^3^; ETOAs: 66–75 cm^3^), albeit with varying distances from the target (EETPA: 12 cm; ETOAs: 11 cm). In contrast, the working volume for MTAs expanded in proportion to the craniotomy size (FTOZAA: 63 cm^3^; RSA: 25 cm^3^). The average distance from the target was shorter than that of the endoscopic approaches (9 cm). Refer to Table 9 for a summary of the minimum, mean, maximum, and standard deviation values pertaining to the non-crossing volume of each simulated approach, with a visual representation provided in Figure 8.

## 4. Discussion

In this anatomical pre-clinical study, we quantitatively compared the percentages of exposure of eight different surfaces of Meckel’s cave by 15 surgical approaches. The experimental findings can be summarized into three main results: (1) the TS is mainly exposed by the RSA; (2) the STA and EETPA can both efficiently expose the GG but the need for major parenchymal retraction must be considered in the microsurgical approach; (3) the EETPA and ETOAs can provide adequate exposure of the most medial compartments of Meckel’s cave, especially for the trigeminal branches, while the MTAs seem to offer the greatest surgical exposure of the lateral compartment of Meckel’s cave. Our data furthermore show clearly how moving anteriorly along the petrous part of the temporal bone posterior approaches causes a loss of exposure power, while that for anterior ones increases.

The existing literature contains a scarcity of quantitative anatomical investigations. These studies have primarily focused on comparing a small selection of surgical approaches to Meckel’s cave, often neglecting the full spectrum of available options and occasionally failing to comprehensively analyze the extent of exposure within the surgical field [15,16,17].

Beyond the anatomical factors, when translating these preclinical findings into a clinical context, it is imperative to remain cognizant of the inherent advantages and disadvantages associated with each surgical approach. Our results are useful for the management of tumors involving Meckel’s cave. These closely related anatomical regions remain a formidable challenge for today’s skull base surgeons due to the intricate bone structures and the presence of critical neurovascular elements that converge within these areas [18,19,20].

Trigeminal schwannomas can present in three different anatomical situations [19,21,22,23]. (1) Schwannomas that involve the trigeminal branches and extend to the pterygopalatine or infratemporal fossae. In this case, the best surgical approach seems to be the EETPA with surface exposure of the medial part of V1, V2, and V3 of 73.9%, 91.3%, and 50.3%; the GG is also well reached by this approach, with surface exposure of 47.4%. This approach is a minimally invasive technique that provides direct access to the pterygopalatine and infratemporal fossae. It has gained popularity in recent years due to its reduced morbidity and faster recovery times [24]. (2) Schwannomas involving only the middle cranial fossa. These tumors grow laterally and medially, pressing Meckel’s cave. In this case, the best surgical approach seems to be the STA with surface exposure of 43.9%. It is interesting to note the gain of exposed surface with brain retraction of 15 mm instead of 10 mm (64.2% vs. 43.9%). This allows the surgeon to carefully evaluate the balance between the benefits and risks of parenchymal retraction, knowing that he will obtain a significant gain in terms of surgical exposure. (3) Trigeminal schwannomas with extension to the TS and/or invasion of the posterior fossa. In this case, the best surgical approaches are KWA or RSAS. KWA is a highly complex but essential middle fossa approach, able to serve a wide array of pathologies together with its extensions. It is very accurate in performing hearing preservation surgery, but not without caveats and an inherent risk of complications [25]. RSAS provides greater exposure of the brainstem and petroclival areas, according to our findings (82.3%) but also according to the literature [15,16].

The KWA is ideally suited for lesions around Meckel’s cave involving the TS but with a main extension into the middle fossa. The KWA exposes significantly less ventral brainstem area than RSAS, as previous studies have confirmed [15]. The mean petroclival area of exposure through the KWA was significantly smaller than that obtained through the RSAS. However, these approaches can be used in conjunction with one another to access petroclival tumors [25]. While trigeminal schwannomas are quite rare, meningiomas are the most frequent Meckel’s cave tumors [26].

Traditionally, three surgical approaches have been described to remove Meckel’s cave meningioma: the STA, the RSA, and the KWA [27]. Still, endoscopic approaches are increasingly used [28], above all when tumors are located anteriorly at the cavernous sinus apex. Biopsy can be performed with EETPA when the percutaneous approach fails, but it also allows tumor removal during the same procedure if indicated. According to our results, EETPA can expose a wide portion of the GG and most of the medial portions of the three trigeminal branches, being particularly useful for small tumors that are located in the anterior portion of Meckel’s cave and that are not associated with significant compression of the trigeminal nerve or other adjacent structures, as Kassam [29] and Jouanneau [28] previously described. For meningiomas located posteriorly in the petrous apex extending to the cerebellopontine angle, without expanding the upper and lower quadrangular spaces of the sphenoid, as described by Cavallo [30], the KWA or RSA is more appropriate.

We found particularly interesting also the trans-orbital approaches, recently described in clinical practice, both as single approaches and combined with EETPA [31,32]. Previous studies have proposed ILTEA as a minimally invasive surgical approach that provides access to the anterior and middle cranial fossae, the cavernous sinus, and the petrous apex [32,33,34]. According to our results, ILTEA can expose wide portions of the lateral parts of V1 and V3 (68.2% and 53.7%) but can reach also the GG with 27.1% of exposure. ILTEAs should be considered as an additional tool rather than a replacement for EETPA or external approaches, to optimize visualization and maneuverability, especially for multicompartmental lesions with extension to the cavernous sinus and petrous apex. SEYA can be used to target lesions involving the anterolateral skull base, as previously described [31].

As far as lesions with parasellar extension are concerned, however, the approach to be preferred is undoubtedly EETPA, given that it allows a wide range of exposure of all the sellar and parasellar regions, as already reported in the literature [35,36,37,38,39,40,41]. To obtain a general overview from the analysis of our anatomical results, it is possible to state that for lesions that grow medially and displace Meckel’s cave laterally, it appears more convenient to perform EETPA, while, for lesions that grow lateral to Meckel’s cave and cause therefore medial compression, it is more appropriate to perform one of the MTAs; if the lesions develop laterally but also present medial involvement, then it may be appropriate to add ILTEA to EETPA.

Our study has several limitations. This was an experimental preclinical investigation, and, as such, it did not consider any distortions in intracranial anatomy, such as the mass effect of the tumor or CSF diversion, when conducting measurements. Additionally, it is important to note that fixation tends to make tissues less flexible and more rigid, potentially resulting in a decreased area of surgical exposure for both endoscopic and transcranial approaches.

## 5. Conclusions

The endoscopic approaches, through the endonasal and transorbital routes, can provide adequate exposure of Meckel’s cave, especially for its more medial portions, bypassing the impediment of major neurovascular structures and significant brain retraction. As far as the most lateral portion of Meckel’s cave, MTAs still seem to be the gold standard in obtaining optimal exposure and adequate surgical volumes. Although limited to a preclinical setting, these findings can provide a valuable contribution to everyday neurosurgical practice and aid in the selection of the most accurate surgical approach to Meckel’s cave.

## Figures and Tables

**Table 1:** Comparison of surgical exposure for GG.

Gasserian Ganglion (GG)		
	% (95% CI)	
	Non-crossing	Crossing
**EETPA**	41.8 (39.3, 45.8)	47.4 (42.6, 50.6)
**FTOZA (10 mm)**	0	0
**FTOZA (15 mm)**	18.6 (4.6, 12.5)	24.5 (15.4, 31.6)
**ILTEA**	22.4 (17.8, 25.9)	27.1 (18.3, 32.5)
**KWA (10 mm)**	2.1 (1.2, 4.5)	4.8 (3.2, 5.1)
**KWA (15 mm)**	3.2 (1.8, 4.8)	6.3 (3.2, 7.8)
**PTA (10 mm)**	0	0
**PTA (15 mm)**	0	0
**RSA (10 mm)**	0	0
**RSA (15 mm)**	0	0
**RSAS (10 mm)**	0	0
**RSAS (15 mm)**	0	0
**SEYA**	5.6 (4.6, 6.0)	6.7 (5.8, 7.5)
**STA (10 mm)**	35.3 (29.5, 39.4)	43.9 (33.4, 47.9)
**STA (15 mm)**	49.7 (42.5, 53.6)	64.2 (51.5, 70.4)

Abbreviations: CI, confidential interval; EETPA, endoscopic endonasal transpterygoid approach; FTOZA, fronto-temporal-orbito-zygomatic approach; ILTEA, infero-lateral transorbital endoscopic approach; RSA, retrosigmoid approach; RSAS, retrosigmoid approach with suprameatal extension; PTA, pterional approach; SEYA, superior eyelid approach; KWA, Kawase approach; STA, subtemporal approach.

**Table 2:** Comparison of surgical exposure for TS.

Trigeminal Stem (TS)		
	% (95% CI)	
	Non-crossing	Crossing
**EETPA**	8.0 (1.2, 12.9)	11.5 (3.4, 17.3)
**FTOZA (10 mm)**	0	0
**FTOZA (15 mm)**	0	0
**ILTEA**	0	0
**KWA (10 mm)**	33.0 (28.2, 35.7)	36.5 (31.2, 42.8)
**KWA (15 mm)**	46.3 (42.3, 52.1)	55.2 (39.2, 65.6)
**PTA (10 mm)**	0	0
**PTA (15 mm)**	0	0
**RSA (10 mm)**	58.1 (55.3, 62.7)	61.4 (60.3, 63.5)
**RSA (15 mm)**	68.6 (65.9, 71.9)	73.2 (69.8, 78.8)
**RSAS (10 mm)**	74.5 (68.2, 78.5)	78.0 (64.1, 82.0)
**RSAS (15 mm)**	78.2 (67.6, 81.7)	82.3 (78.6, 85.4)
**SEYA**	0	0
**STA (10 mm)**	0	0
**STA (15 mm)**	0	0

Abbreviations: CI, confidential interval; EETPA, endoscopic endonasal transpterygoid approach; FTOZA, fronto-temporal-orbito-zygomatic approach; ILTEA, infero-lateral transorbital endoscopic approach; RSA, retrosigmoid approach; RSAS, retrosigmoid approach with suprameatal extension; PTA, pterional approach; SEYA, superior eyelid approach; KWA, Kawase approach; STA, subtemporal approach.

**Table 3:** Comparison of surgical exposure for V1m.

V1 Medial (V1m)		
	% (95% CI)	
	Non-crossing	Crossing
**EETPA**	66.3 (52.1, 74.2)	73.9 (61.6, 88.5)
**FTOZA (10 mm)**	0	0
**FTOZA (15 mm)**	0	0
**ILTEA**	0	0
**KWA (10 mm)**	0	0
**KWA (15 mm)**	0	0
**PTA (10 mm)**	0	0
**PTA (15 mm)**	0	0
**RSA (10 mm)**	0	0
**RSA (15 mm)**	0	0
**RSAS (10 mm)**	0	0
**RSAS (15 mm)**	0	0
**SEYA**	0	0
**STA (10 mm)**	0	0
**STA (15 mm)**	0	0

Abbreviations: CI, confidential interval; EETPA, endoscopic endonasal transpterygoid approach; FTOZA, fronto-temporal-orbito-zygomatic approach; ILTEA, infero-lateral transorbital endoscopic approach; RSA, retrosigmoid approach; RSAS, retrosigmoid approach with suprameatal extension; PTA, pterional approach; SEYA, superior eyelid approach; KWA, Kawase approach; STA, subtemporal approach.

**Table 4:** Comparison of surgical exposure for V1l.

V1 Lateral (V1l)		
	% (95% CI)	
	Non-crossing	Crossing
**EETPA**	5.3 (2.1, 6.2)	6.1 (3.6, 8.5)
**FTOZA (10 mm)**	89.4 (84.5, 93.1)	93.4 (88.5, 98.4)
**FTOZA (15 mm)**	93.8 (81.8, 95.9)	96.2 (91.3, 98.4)
**ILTEA**	60.7 (48.5, 72.4)	68.2 (56.3, 76.8)
**KWA (10 mm)**	0	0
**KWA (15 mm)**	0	0
**PTA (10 mm)**	23.5 (44.6, 57.1)	36.1 (49.0, 59.9)
**PTA (15 mm)**	27.1 (47.8, 58.3)	39.2 (53.2, 61.3)
**RSA (10 mm)**	0	0
**RSA (15 mm)**	0	0
**RSAS (10 mm)**	0	0
**RSAS (15 mm)**	0	0
**SEYA**	2.3 (1.2, 3.4)	2.9 (1.2, 3.5)
**STA (10 mm)**	40.3 (34.2, 48.9)	51.9 (41.0, 63.8)
**STA (15 mm)**	45.9 (35.8, 54.1)	57.6 (44.2, 69.2)

Abbreviations: CI, confidential interval; EETPA, endoscopic endonasal transpterygoid approach; FTOZA, fronto-temporal-orbito-zygomatic approach; ILTEA, infero-lateral transorbital endoscopic approach; RSA, retrosigmoid approach; RSAS, retrosigmoid approach with suprameatal extension; PTA, pterional approach; SEYA, superior eyelid approach; KWA, Kawase approach; STA, subtemporal approach.

**Table 5:** Comparison of surgical exposure for V2m.

V2 Medial (V2m)		
	% (95% CI)	
	Non-crossing	Crossing
**EETPA**	83.1 (75.3, 92.6)	91.3 (82.7, 96.0)
**FTOZA (10 mm)**	0	0
**FTOZA (15 mm)**	0	0
**ILTEA**	1.6 (1.0, 2.5)	5.3 (2.4, 6.8)
**KWA (10 mm)**	0	0
**KWA (15 mm)**	0	0
**PTA (10 mm)**	0	0
**PTA (15 mm)**	0	0
**RSA (10 mm)**	0	0
**RSA (15 mm)**	0	0
**RSAS (10 mm)**	0	0
**RSAS (15 mm)**	0	0
**SEYA**	0	0
**STA (10 mm)**	0	0
**STA (15 mm)**	0	0

Abbreviations: CI, confidential interval; EETPA, endoscopic endonasal transpterygoid approach; FTOZA, fronto-temporal-orbito-zygomatic approach; ILTEA, infero-lateral transorbital endoscopic approach; RSA, retrosigmoid approach; RSAS, retrosigmoid approach with suprameatal extension; PTA, pterional approach; SEYA, superior eyelid approach; KWA, Kawase approach; STA, subtemporal approach.

**Table 6:** Comparison of surgical exposure for V2l.

V2 Lateral (V2l)		
	% (95% CI)	
	Non-crossing	Crossing
**EETPA**	8.1 (2.9, 15.4)	13.5 (12.0, 21.8)
**FTOZA (10 mm)**	19.1 (14.4, 26.0)	23.7 (18.5, 30.9)
**FTOZA (15 mm)**	30.6 (21.8, 38.5)	39.8 (31.6, 44.5)
**ILTEA**	28.6 (21.0, 34.6)	35.3 (26.4, 46.1)
**KWA (10 mm)**	0	0
**KWA (15 mm)**	0	0
**PTA (10 mm)**	3.1 (1.5, 5.6)	5.0 (3.8, 9.5)
**PTA (15 mm)**	5.4 (3.8, 8.3)	9.2 (6.6, 11.9)
**RSA (10 mm)**	0	0
**RSA (15 mm)**	0	0
**RSAS (10 mm)**	0	0
**RSAS (15 mm)**	0	0
**SEYA**	0	0
**STA (10 mm)**	44.7 (35.6, 52.9)	60.1 (51.8, 71.0)
**STA (15 mm)**	57.9 (49.1, 64.3)	72.4 (58.2, 79.3)

Abbreviations: CI, confidential interval; EETPA, endoscopic endonasal transpterygoid approach; FTOZA, fronto-temporal-orbito-zygomatic approach; ILTEA, infero-lateral transorbital endoscopic approach; RSA, retrosigmoid approach; RSAS, retrosigmoid approach with suprameatal extension; PTA, pterional approach; SEYA, superior eyelid approach; KWA, Kawase approach; STA, subtemporal approach.

**Table 7:** Comparison of surgical exposure for V3m.

V3 Medial (V3m)		
	% (95% CI)	
	Non-crossing	Crossing
**EETPA**	41.9 (35.7, 52.6)	50.3 (46.5, 63.0)
**FTOZA (10 mm)**	0	0
**FTOZA (15 mm)**	0	0
**ILTEA**	11.2 (9.0, 16.4)	15.8 (11.1, 20.6)
**KWA (10 mm)**	0	0
**KWA (15 mm)**	0	0
**PTA (10 mm)**	0	0
**PTA (15 mm)**	0	0
**RSA (10 mm)**	0	0
**RSA (15 mm)**	0	0
**RSAS (10 mm)**	0	0
**RSAS (15 mm)**	0	0
**SEYA**	2.3 (1.9, 5.5)	7.1 (4.4, 10.9)
**STA (10 mm)**	0	0
**STA (15 mm)**	0	0

Abbreviations: CI, confidential interval; EETPA, endoscopic endonasal transpterygoid approach; FTOZA, fronto-temporal-orbito-zygomatic approach; ILTEA, infero-lateral transorbital endoscopic approach; RSA, retrosigmoid approach; RSAS, retrosigmoid approach with suprameatal extension; PTA, pterional approach; SEYA, superior eyelid approach; KWA, Kawase approach; STA, subtemporal approach.

**Table 8:** Comparison of surgical exposure for V3l.

V3 Lateral (V3l)		
	% (95% CI)	
	Non-crossing	Crossing
**EETPA**	0.5 (0, 1.3)	3.6 (2.2, 6.3)
**FTOZA (10 mm)**	29.6 (24.5, 36.9)	37.3 (28.9, 48.2)
**FTOZA (15 mm)**	42.4 (30.0, 47.4)	49.1 (35.6, 56.0)
**ILTEA**	44.9 (31.8, 53.7)	53.7 (42.1, 58.5)
**KWA (10 mm)**	0	0
**KWA (15 mm)**	0	0
**PTA (10 mm)**	13.6 (8.2, 17.9)	15.3 (10.1, 21.5)
**PTA (15 mm)**	25.7 (13.6, 31.4)	31.8 (26.8, 42.3)
**RSA (10 mm)**	0	0
**RSA (15 mm)**	0	0
**RSAS (10 mm)**	0	0
**RSAS (15 mm)**	0	0
**SEYA**	17.0 (11.8, 23.6)	25.8 (12.4, 31.9)
**STA (10 mm)**	56.5 (41.3, 62.7)	65.6 (60.9, 77.4)
**STA (15 mm)**	64.6 (54.2, 76.9)	73.6 (64.8, 81.9)

Abbreviations: CI, confidential interval; EETPA, endoscopic endonasal transpterygoid approach; FTOZA, fronto-temporal-orbito-zygomatic approach; ILTEA, infero-lateral transorbital endoscopic approach; RSA, retrosigmoid approach; RSAS, retrosigmoid approach with suprameatal extension; PTA, pterional approach; SEYA, superior eyelid approach; KWA, Kawase approach; STA, subtemporal approach.

**Table 9:** Table featuring the minimum, mean, maximum, and standard deviation values, measured in cubic centimeters (cm^3^), for the non-crossing volume in each simulated approach.

Approach	Average	Minimum	Maximum	Standard Deviation
**EETPA**	84.7	68.1	95.3	9.6
**FTOZA**	62.9	56.4	77.2	5.4
**ILTEA**	75.4	66.2	86.8	8.2
**KWA**	35.6	26.9	44.3	4.2
**PTA**	35.5	29.2	46.7	4.0
**RSA**	25.1	20.7	33.9	3.7
**RSAS**	30.4	21.0	38.5	3.9
**SEYA**	66.3	46.0	75.5	7.3
**STA**	33.1	27.4	41.8	3.9

Abbreviations: EETPA, endoscopic endonasal transpterygoid approach; FTOZA, fronto-temporal-orbito-zygomatic approach; ILTEA, infero-lateral transorbital endoscopic approach; RSA, retrosigmoid approach; RSAS, retrosigmoid approach with suprameatal extension; PTA, pterional approach; SEYA, superior eyelid approach; KWA, Kawase approach; STA, subtemporal approach.

## Data Availability

Data available in a publicly accessible repository.

## Abbreviations

CI = confidential interval; EETPA = endoscopic endonasal transpterygoid approach; ETOA = endoscopic transorbital approach; FTOZA = fronto-temporal-orbito-zygomatic approach; GG = Gasserian ganglion; ILTEA = infero-lateral transorbital endoscopic approach; KWA = Kawase approach; MTA = microsurgical transcranial approach; RSA = retrosigmoid approach; RSAS = retrosigmoid approach with suprameatal extension; PTA = pterional approach; SEYA = superior eyelid approach; TS = trigeminal stem; STA = subtemporal approach; V1l = lateral ophthalmic nerve; V1m = medial ophthalmic nerve; V2m = medial maxillary nerve; V2l = lateral maxillary nerve; V3m = medial mandibular nerve; V3l = lateral mandibular nerve.
